# The Medical News Visiting List

**Published:** 1890-12

**Authors:** 


THE MEDICAL NEWS VISITING LIST for 1891. Weekly (dated for thirty patients) ; Monthly (undated, for 120 patients per month) ; Perpetual (undated, for thirty patients weekly per pear) ; and Perpetual (undated, for sixty patients weekly per year). The first three styles contain 32 pages of data and 176 pages of blanks. The 60-Patient Perpetual consists of 256 pages of blanks. Each style in one wallet-shaped book, pocket, pencil, rubber, erasible tablet, etc. Leather, $1.25. Philadelphia : Lea Brothers & Co. 1890.

This popular visiting list, now making its annual tour, has been thoroughly revised and brought up to date in every respect. The text portion (thirty-two pages) contains the most useful data for the physician and surgeon, including an alphabetical Table of Diseases, with the most approved remedies, and a Table of Doses, both prepared from Dr. H. A. Hare's new *Text-Book of Practical Therapeutics.* It also contains sections on Examination of Urine, Artificial Respiration, Incompatibles, Poisons and Antidotes, Diagnostic Table of Eruptive Fevers, Ligation of Arteries, and a full descriptive list of valuable remedies not yet in general use. The classified blanks (176 pages) are arranged to hold records of all kinds of professional work, with memoranda and accounts. Four styles are now published : Weekly (dated for thirty patients) ; Monthly (undated for 120 patients per month, and good for any year) ; Perpetual (undated, for thirty patients weekly per year) ; and Perpetual (undated, for sixty patients weekly per year). This last style, a novelty for the coming year, consists of 256 pages of assorted record blanks, without text. *The Medical News Visiting List* adapts itself to any system of keeping professional accounts. Each style is in one volume, bound in handsome red leather, with pocket, pencil, rubber, erasible tablet and catheter-scale ; price, $1.25. When desired, a ready reference thumb-letter index is furnished, which is peculiar to this visiting list, and will save many-fold its small cost (25 cents) in the economy of time effected during a year. In short, every need of the physician seems to have been anticipated in *The Medical News Visiting List.*

